# Contact Lens Visual Rehabilitation in Keratoconus and Corneal Keratoplasty

**DOI:** 10.1155/2012/832070

**Published:** 2012-01-15

**Authors:** Yelda Ozkurt, Mehmet Atakan, Tugba Gencaga, Sezen Akkaya

**Affiliations:** ^1^Department of Opthalmology, FSM Training and Research Hospital, Icerenkoy, Istanbul, Turkey; ^2^Department of Opthalmology, Dr. Lutfi Kirdar Training and Research Hospital, Kartal, Istanbul, Turkey

## Abstract

Keratoconus is the most common corneal distrophy. It's a noninflammatory progressive thinning process that leads to conical ectasia of the cornea, causing high myopia and astigmatism. Many treatment choices include spectacle correction and contact lens wear, collagen cross linking, intracorneal ring segments implantation and finally keratoplasty. Contact lenses are commonly used to reduce astigmatism and increase vision. There are various types of lenses are available. We reviewed soft contact lenses, rigid gas permeable contact lenses, piggyback contact lenses, hybrid contact lenses and scleral-semiscleral contact lenses in keratoconus management. The surgical option is keratoplasty, but even after sutur removal, high astigmatism may stil exists. Therefore, contact lens is an adequate treatment option to correct astigmatism after keratoplasty.

## 1. Introduction

Keratoconus is a Greek word (kerato: cornea; konos: cone) meaning cone-shaped protrusion of the cornea. Keratoconus is a condition with noninflammatory, progressive thinning and steepening of the central and/or paracentral cornea. It is the most common primary ectasia and usually occurs in the second decade of life and affects both genders and all ethnicities. The estimated prevalence in the general population is 54 per 100,000 [1].

Etiology is unknown and most likely multifactorial. Recent research suggests that keratoconus somehow accelerates the process of keratocyte apoptosis, which is the programmed death of corneal cells that occurs following injury. Minor external traumas, such as poorly fitted contact lenses, ocular allergies [2], and eye rubbing mostly due to atopy [3] can release cytokines from the epithelium that stimulate keratocyte apoptosis. Early studies demonstrated elevated levels of collagenolytic and gelatinolytic activities in keratoconic corneas. Although thought to be a non-inflammatory disease, inflammatory molecules, such as interleukins and tumor necrosis factor, have been shown to be elevated in keratoconus, and these inflammatory molecules may mediate production and activation of proteases [4]. Genetics may play a role in the etiology of keratoconus, in that some patients may have a genetic predisposition [5]. Genetic heterogeneity consists of allelic heterogeneity (different mutations in the same locus) and/or locus heterogeneity with different loci producing the same phenotype. To date, locus heterogeneity has been extensively observed in KTCN studies. Linkage analysis and association studies are the two main approaches used to identify the causative genes. Linkage analysis identifies chromosomal region(s) associated with the disease and the gene(s) mapped to that regions [6] In complex disease, where more than one gene is considered, gene-gene interaction should also be investigated. One of the attempts to present the disease more realistically in a linkage analysis is a method allowing for analyzing two distinct loci simultaneously. Such analysis performed in an Australian pedigree by Burdon et al., identified 1p36.23–36.21 and 8q13.1–q21.11 loci [7]. To date, only one keratoconus locus, 5q21.2, previously reported by Tang et al. [8] has been replicated by Bisceglia et al.

## 2. Familial Keratoconus

Although the majority of patients presenting to ophthalmologists with keratoconus have a sporadic form of the disease, there is growing evidence of familial keratoconus and the involvement of genetic factors [9]. Ninety percent of pedigrees with familial keratoconus display an autosomal dominant inheritance with reduced penetrance [10]. Numerous loci have been mapped in keratoconus families, and research is ongoing to identify causative genes involved in keratoconus development and progression, such as a locus for autosomal dominant keratoconus was mapped in Finnish families to 16q22.3–q23.1 [11]. More than two dozen syndromes are associated with keratoconus, including Down syndrome, connective tissue disorders, including osteogenesis imperfecta, and some subtypes of Ehlers-Danlos syndrome [12]. The complexity of keratoconus makes it difficult to identify factors influencing its development. Identification of genetic factors might allow to develop both specific diagnostic tests and keratoconus gene therapy in the future.

Keratoconus can involve each layer of the cornea. Early degeneration of basal epithelial cells can be followed by disruption of the basement membrane. The stroma has normal-sized collagen fibers but low numbers of collagen lamellae, which results in stromal thinning. The irregular superficial opacities and scars at or near the apex of the cone represent structural breaks in Bowman's layer. Vogt's striae are fine, and parallel striations stress lines of the stroma might be present. Moreover, cornea demonstrated endothelial cell pleomorphism and polymegathism and endothelial cell degeneration [13]. Finally if there is a spontaneous tear in Descemet's membrane, aqueous flows into stroma creates acute corneal edema called “hydrops.”

## 3. Contact Lens in Keratoconus

Soft contact lenses have limited role in correcting corneal irregularity, as they tend to drape over the surface of the cornea and result in poor visual acuity. Early in the disease, soft lenses with toric design may be adequate to correct myopia and regular astigmatism. However, soft lenses designed *specifically *for keratoconus (e.g., KeraSoft) have a useful role in early keratoconus or where a patient may be intolerant of RGP. Soft lenses tend to be more comfortable compared with RGPs. Rigid gas permeable (RGP) lenses are required as the condition progresses in order to correct the irregular astigmatism. The aim is to provide the best vision possible with the maximum comfort. All keratoconus contact lenses should be ordered in a moderate to high Dk rigid gas permeable material to avoid epithelial hypoxia and corneal erosion during the long wearing schedule of keratoconus patient. These lenses have different fitting types.

- The three-point-touch design is the most popular and the most widely fitted design for keratoconic patients. Three-point-touch actually refers to the area of apical central contact and two other areas of bearing or contact at the midperiphery in the horizontal direction [14].
- Apical clearance: in this type of fitting technique, the lens vaults the cone and clears the central cornea, resting on the paracentral cornea. The potential advantages are reducing central corneal scarring. However, the disadvantages are causing a poor tear film, corneal oedema, and poor visual acuity as a result of bubbles under the lens.
- The apical bearing technique: the weight of the lens is supported by the area on the apex of the cornea but not elsewhere on the cornea. The advantage of this fit is that patients may have good visual acuity obtained as a result of apical touch. But it also may accelerate the corneal scarring due to touch [15].

In some keratoconic patients, the steepness of the corneal apex and the radical flattening of the mid-peripheral and peripheral cornea limit the effective use of spherical lenses to correct irregularity. An aspheric lens with a high eccentricity value will become flatter quicker compared to a spherical curve. This allows you to select a relatively steep base curve radius to match the apex of the cornea and the highly aspheric posterior designs provide better alignment and weight distribution over a larger area of the cornea. This often provides improved lens centration and comfort. The aim of aspheric lens fit should be good centration, central alignment or slight central bearing, good movement (1 mm), and peripheral clearance. There are various types of lenses with monocurve or multicurve design.

*The McGuire System*: The McGuire system was first introduced in 1978 and consists of three diagnostic lens sets, nipple, oval, or globus. McGuire system has four peripheral curves that make the lens easy to fit [16].

*The Rose K* is a unique keratoconus lens design with complex computer-generated peripheral curves based on data collected by Dr. Paul Rose of Hamilton, New Zealand. The system (26 lens set) incorporates a triple peripheral curve system [17, 18].

*Piggyback Lenses* are used for difficult cases, for instance in cases of RGP lens intolerance, proud nebulae in keratoconus, or apical dimpling or where there are areas of recurrent epithelial erosion. The system consists of a rigid lens fitted on top of a soft lens aiming to obtain same visual acuity as with a single lens. Soft lens must be a silicone hydrogel lens with a high Dk/t [19].

*Hybrid Lens System*; The Softperm lens (Ciba Vision) is a hybrid lens with a RGP centre surrounded by a soft hydrophilic skirt. The SynergEyes is relatively new and with a high Dk hybrid lens, it could be used for early keratoconus due to its aspherical design. These lenses tend to be used in cases of RGP lens intolerance. A recent study performed by Abdalla et al. demonstrated that such RGP intolerant patients showed great optical improvement with this hybrid lens [20]. But the main limitations are giant papillary conjunctivitis and peripheral vascularization.

Scleral and Semiscleral Lenses have proven to be extremely beneficial for patients with highly irregular and/or asymmetric keratoconic corneas. These patients will benefit from a large diameter (13.5 to 16.0 mm) semiscleral lens design. Schornack et al. showed a dramatic improvement in visual acuity by using scleral lens in a study [21, 22].

## 4. Contact Lens following Keratoplasty

For keratoconus surgery might be considered when patients are no longer able to tolerate their gas-permeable contact lenses, when a successful contact lens fit is no longer possible or because of unresolving corneal hydrops. Penetrating keratoplasty* (PK) *or full-thickness corneal transplant, historically has been the most common surgical correction for irregular astigmatism resulting from keratoconus. The corneal graft is susceptible to epithelial, stromal, and endothelial forms of inflammatory rejection from the host's immune response. The purpose of deep anterior lameller keratoplasty* (DALK) *is to preserve host Descemet's membrane and endothelium. This may decrease overall graft rejection episodes, including stromal and epithelial rejection.

The main reason of decreased visual acuity after keratoplasty is most likely high astigmatism. Generally, even after suture removal, residual astigmatism still cause visual problems. Various types of treatment modalities are tried with contact lenses: sclerals, rigid gas permeable and reverse geometry hydrogel lenses, and silicone hydrogel soft toric contact lenses, in order to improve lens and optical stability, but no common consensus is approved yet [23–25].

If postresidual astigmatism is under 4D, a hard gas permeable, large-diametered contact lens will be recommended, bearing in mind that donor cornea diameter should be smaller than contact lens diameter, eventually. If astigmatism is under 1D, soft contact lenses would be successful to correct refractive status. In a recent study, Geerards et al. successfully fitted large-diameter (12 mm) tricurve rigid gas-permeable contact lenses for 90 (47%) of 190 penetrating keratoplasty patients with good tolerance [26]. Intralimbal rigid gas-permeable contact lenses are found effective in increasing visual acuity after penetrating keratoplasty, keratoconus and pellucid marginal degeneration as well [27]. Also special design contact lenses can improve visual acuity after penetrating keratoplasty. Gruenauer-Kloevekorn et al. fitted 4 different types of special contact lenses in 28 eyes, and nearly in all patients visual acuity significantly improved [28]. In conclusion, there are many contact lens options available to correct postkeratoplasty astigmatism before conducting any surgical method.
